# Effect of a GH Secretagogue, Anamorelin, on Serum Irisin and Inflammation Levels in Osteosarcopenic Adults

**DOI:** 10.1210/jendso/bvae028

**Published:** 2024-02-13

**Authors:** Bess Dawson-Hughes, Elsa M Konieczynski, Elise Reitshamer, Lisa Ceglia

**Affiliations:** Bone Metabolism Laboratory, Jean Mayer USDA Human Nutrition Research Center on Aging at Tufts University, Boston, MA 02108, USA; Bone Metabolism Laboratory, Jean Mayer USDA Human Nutrition Research Center on Aging at Tufts University, Boston, MA 02108, USA; Bone Metabolism Laboratory, Jean Mayer USDA Human Nutrition Research Center on Aging at Tufts University, Boston, MA 02108, USA; Bone Metabolism Laboratory, Jean Mayer USDA Human Nutrition Research Center on Aging at Tufts University, Boston, MA 02108, USA; Endocrine Division, Tufts Medical Center, Boston, MA 02108, USA

**Keywords:** irisin, IGF-1, IL-6, hsCRP, osteosarcopenia

## Abstract

**Background:**

Data suggest an association between GH secretion and circulating levels of the myokine irisin and inflammatory cytokinesIL-6 and high-sensitivity C-reactive protein (hsCRP). The impact of GH secretagogues on these markers is unknown.

**Objectives:**

To determine the effect of treatment with the GH secretagogue anamorelin on 12-month changes in serum irisin, IL-6, and hsCRP levels and to assess whether baseline irisin levels modulate the glycemic response to treatment with anamorelin.

**Methods:**

This is an ancillary study in 26 older adults with osteosarcopenia who participated in a 12-month trial examining the effect of anamorelin 100 mg/day vs placebo on musculoskeletal outcomes. Serum irisin, IL-6, and hsCRP were measured at baseline and 12 months.

**Results:**

Treatment with anamorelin, compared with placebo, did not significantly alter irisin levels [12-month change = 0.50 ± 1.2 (SD) ng/mL in anamorelin group and −0.08 ± 2.3 ng/mL in placebo; *P* = .191]. Baseline irisin levels were not significantly correlated with 2-month change in fasting glucose levels in the anamorelin group (r = −0.222, *P* = .46) or the placebo group (r = 0.30, *P* = .34); however, the slopes of the 2 regression lines describing the relationship by group tended to differ (*P* = .0547). Anamorelin treatment for 12 months had no significant effect on serum IL-6 or hsCRP levels.

**Conclusion:**

In this small sample of older adults with osteosarcopenia, treatment with the GH secretagogue anamorelin did not significantly alter levels of irisin, IL-6, or hsCRP. Higher baseline irisin levels may attenuate the glycemic response to anamorelin treatment; however, a larger study is needed to confirm this possibility.

Irisin is a small peptide secreted from muscle in response to muscle contraction. It was first described by Boström in 2012 as a potential myokine that stimulates the conversion of white fat to brown fat [1]. Irisin has since been purported to have an impressive array of other roles, including preventing muscle atrophy [2], increasing osteoblast differentiation [3], and reducing the prevalence of osteoporotic fractures [4]. It has also been proposed as a senolytic agent [5].

Irisin may play a role in mediating the effect of the growth GH/IGF-1 axis on its target tissues. Acromegalic patients have been reported to have elevated irisin levels compared to normal controls [6], and GH treatment in deficient children significantly increased their irisin levels [7]. However, current evidence is inconsistent as illustrated in another study in which adults being treated for acromegaly had lower irisin levels than controls [8]. As noted by Calan [6], increased irisin levels in patients with active acromegaly could arise either as a consequence of the GH excess or in response to the unfavorable metabolic changes (eg, insulin resistance) that accompany GH excess. Higher circulating irisin levels may ameliorate glycemia by stimulating glucose uptake into skeletal muscle cells [9].

GH may inhibit the expression of proinflammatory cytokines. GH replacement therapy in patients with hypopituitarism has reduced inflammatory markers including IL-6 [10] and C-reactive protein [11, 12], although there is some opposing evidence, as reviewed by Szalecki et al [13]. It is unknown whether treatment with a GH secretagogue would affect inflammatory cytokines in populations with a modest deficiency in GH secretion such as older adults with osteosarcopenia.

We recently completed a small 12-month randomized controlled trial to assess the effect of the GH secretagogue and the ghrelin receptor agonist anamorelin on musculoskeletal outcomes in adults with osteosarcopenia [14]. In that trial, anamorelin, when compared with placebo, increased the circulating IGF-1 level by 50% and modestly increased the mean fasting plasma glucose level [14]. Anamorelin also increased leg strength and bone formation but had no effect on muscle mass or bone resorption [14]. The objectives of this ancillary study were to assess (1) whether treatment with anamorelin increased the circulating level of irisin, (2) whether the participants with higher baseline irisin levels had an attenuated glycemic response to anamorelin, and (3) whether anamorelin treatment altered the inflammatory markers serum IL-6 and high-sensitivity C-reactive protein (hsCRP).

## Methods

### Subjects and Study Design

Participants were 26 men and women age 50 years and older who completed a 12-month clinical trial designed to assess the effects of the GH secretagogue anamorelin or placebo on bone and muscle outcomes. Participants were recruited from the Boston area. We screened 74 and enrolled 32 participants. Of 15 assigned to placebo, 13 completed the study (1 relocated and 1 lost interest); of 17 assigned to anamorelin, 13 completed the study (2 discontinued because of mild gastrointestinal symptoms and 1 for musculoskeletal aches, and 1 with type 2 diabetes was discontinued because of an elevated fasting plasma glucose level). All participants had osteopenia as defined by the World Health Organization [bone mineral density T-score between −1.0 and −2.5 at the nondominant total hip, femoral neck, or lumbar spine (at L1, L2, L3, or L4) and sarcopenia as defined by the Sarcopenia Definitions and Outcomes Consortium (maximum grip strength <35.5 kg [men] and <20 kg [women] in either hand [excluding hands with severe pain or recent surgery] and/or gait speed <0.8 m/sec)]. Study exclusions included inflammatory disorders, use of insulin or sulfonylureas, and abnormal liver function tests. Additional details are provided elsewhere [14].

In this double-blind, placebo-controlled trial, participants were randomized 1 to 1 (with a block size of size 4) with the use of the randomization module in REDCap. Only the research pharmacist and the study statistician were unmasked. The protocol was approved by the Human Investigation Review Committee of Tufts University (IRB #13390), and written informed consent was obtained from each participant. The study is registered at Clinicaltrials.gov under NCT04021706.

### Laboratory Analyses

Between June 2021 and January 2022, serum samples were collected on baseline, 2-month (glucose only), and 12-month visits after a 12-hour fast; samples were frozen immediately and stored at −80° C. The samples, not previously thawed, were batched for analysis. Irisin was measured with the use of ELISA EK-067-29 kits from Phoenix Pharmaceuticals (Phoenix Pharmaceuticals, cat. no. EK-067-52, RRID:AB_2783013). IL-6 was measured on a Meso Scale Discovery with cat. no. K151QXD-1, RRID:AB_3076416, and serum hsCRP was measured with use of Siemens cat. no. L2K6P2, RRID:AB_2904178. Plasma glucose was measured on an Olympus AU400 clinical chemistry analyzer, and serum IGF-1 was measured by solid-state, two-site chemiluminescent immunometric assay (Siemens, cat. no. L2KGF2, RRID:AB_2756880), as described previously [14].

### Statistical Methods

These secondary analyses were conducted on the 26 participants who completed the study. Irisin measurements are available only in these 26 participants. We examined univariate and bivariate distributions of variables for departures from normality and linearity. Three baseline values >3 SD above the group-specific mean were excluded from the analyses in which they were an outlying value: 1 irisin value in the placebo group, 1 IL-6 value in the anamorelin group, and 1 hsCRP value in the placebo group.

Baseline, 12-month, and 12-month change in biochemical measurements are reported as mean ± SD. Bivariate relationships are described by Pearson correlation coefficients. Group differences in 12-month change in biochemical measurements were assessed by exact two-sample Mann–Whitney test. To evaluate a potential group difference in the relationship between baseline irisin and change in fasting glucose, we used a Wald test to compare the slopes of 2 simple linear regression equations that modeled the relationship separately by group. All statistical analyses were performed in STATA/SE 18.0.

## Results

Participants assigned to placebo (n = 13) and anamorelin (n = 13) had mean ages of 75.5 ± 6.4 (SD) and 73.2 ± 5.8 years and mean body mass indices of 23.7 and 23.8 kg/m^2^, respectively. These and other baseline characteristics did not differ significantly by group [14].

Serum irisin levels did not differ in the 2 treatment groups at baseline (Table 1), and 12-month changes in serum irisin levels did not differ significantly in the 2 groups (Table 1). The serum IGF-1 level increased with anamorelin treatment and was unchanged in the placebo group (Table 1).

**Table 1.** Baseline and 12-month biochemical measurements and their changes, by treatment group

	n	Group	M0	M12	12-month change	*P^a^*
Irisin, ng/mL	12	Placebo	5.93 ± 1.8	5.86 ± 2.0	−0.08 ± 2.3	.199
	13	Anamorelin	5.98 ± 1.5	6.48 ± 1.2	0.50 ± 1.2	
IL-6, pg/mL	13	Placebo	2.39 ± 1.7	1.69 ± 0.8	−0.70 ± 1.7	.210
	12	Anamorelin	1.98 ± 2.0	1.84 ± 1.6	−0.14 ± 1.11	
hsCRP, mg/L	12	Placebo	2.23 ± 1.3	1.75 ± 1.2	−0.48 ± 1.5	.469
	13	Anamorelin	1.88 ± 2.5	2.33 ± 4.4	0.45 ± 4.2	
IGF-1, ng/mL	13	Placebo	110 ± 22	107 ± 38	−2.2 ± 23	.0001
	13	Anamorelin	108 ± 14	162 ± 47	53.5 ± 38	

Abbreviations: hsCRP, high-sensitivity C-reactive protein.

Mean ± SD shown.

^*a*^ *P*-values for irisin, IL-6, and hsCRP are the between-group comparisons, two-sample Mann–Whitney test on 12-month change.

**Figure 1.**
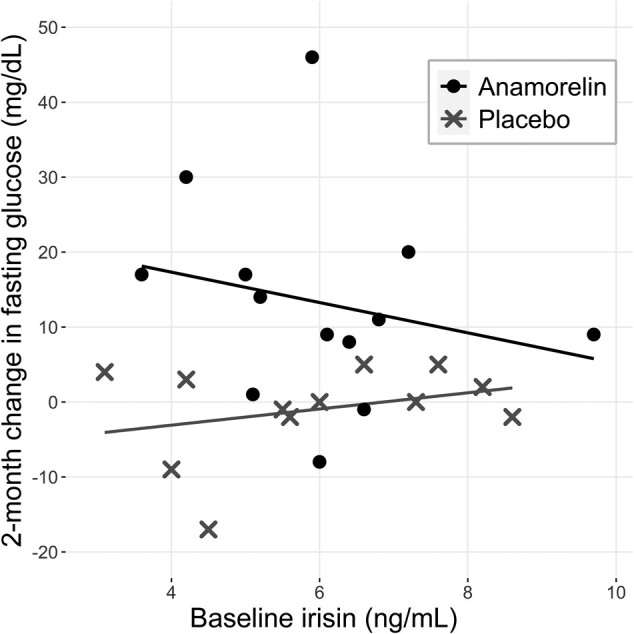
Scatterplots of baseline irisin levels with 2-month change in fasting plasma glucose in the anamorelin (circles) and placebo (crosses) groups. There was a trend toward a difference in slopes of the regression lines. (ß=1.08 in placebo and −2.03 in anamorelin group; *P* = .0547).

Fasting glucose levels were similar in the 2 groups at baseline (100 ± 8 mg/dL in placebo and 95 ± 8 mg/dL in anamorelin); however, the 2-month changes differed (−0.38 ± 6.5 mg/dL in the placebo group and 13.3 ± 13.8 in the anamorelin group, *P* = .002). Baseline irisin levels were not significantly correlated with 2-month change in fasting plasma glucose levels in the anamorelin group (r = −0.222, *P* = .46) or the placebo group (r = 0.30, *P* = .34); however, the slopes of the 2 regression lines tended to differ (ß = 1.08 in placebo and ß = −2.03 in the anamorelin group; *P* = .0547; Fig. 1). Adjustment for PASE physical activity score, a potential determinant of the circulating irisin level, did not alter this association.

Serum IL-6 and hsCRP levels did not differ significantly in the 2 groups at baseline, and their 12-month changes did not differ significantly in the 2 groups (Table 1). There was no significant correlation of baseline IL-6 or hsCRP level with change in IGF-1 in either the anamorelin or placebo group (data not shown), and there was no significant correlation between 12-month change in IGF-1 and 12-month change in either IL-6 (r = 0.264, *P* = .202) or hsCRP (r = 0.128, *P* = .54).

## Discussion

In older adults with osteosarcopenia, treatment for 1 year with the GH secretagogue anamorelin had no significant impact on serum irisin levels, despite the fact that anamorelin increased the circulating IGF-1 level by 50%. Since anamorelin did not significantly alter muscle mass in this study [14], it is not surprising that anamorelin treatment had no apparent effect on the circulating level of irisin, a hormone that arises mainly from muscle tissue. The proposed role for irisin in mediating the effects of the GH/IGF-1 axis on the musculoskeletal system stems from studies of patients with GH excess or deficiency. Calan et al found that irisin levels were significantly increased both in patients with uncontrolled and with controlled acromegaly, when compared with normal controls [6]. They also noted that irisin levels were higher in the uncontrolled than in the controlled acromegalic patients [6]. Additional evidence comes from a study of 54 GH-deficient children, in whom exogenous GH treatment significantly increased both IGF-1 and irisin levels [7]. Moreover, after 12 months of treatment, the irisin and IGF-1 levels in these children were highly correlated (r = 0.865, *P* < .001) [7]. In contrast to these studies indicating a stimulatory effect of the GH/IGF-1 axis on irisin levels, another study in 43 adults with acromegaly, most of whom were controlled, observed that irisin levels were lower in adults with acromegaly than in the controls [8]. Our finding that anamorelin treatment had no impact on serum irisin levels despite its inducing a substantial increase in IGF-1 levels provides direct evidence that the metabolic effects of the GH/IGF-1 axis in adults with osteosarcopenia are unlikely to be mediated through irisin.

We examined whether fasting glucose levels during anamorelin treatment may have been influenced by the circulating irisin level. Boström et al [1] noted that irisin expression in mice fed high-fat diets caused improvement in glucose tolerance. These authors commented that even modestly increased levels of circulating irisin potently increased energy expenditure and decreased diet-induced insulin resistance in the mice. Lee et al exposed muscle cell cultures to irisin and documented a dose-related increase in glucose uptake [9]. Our study confirmed the characteristic, modest increase in fasting glucose levels consistently seen with anamorelin [15, 16] and other GH secretagogues [17, 18]. Our preliminary observation that baseline irisin levels and 2-month changes in glucose were inversely related in the anamorelin group, but not in the placebo group, suggests that irisin may have a role in attenuating the glycemic response to anamorelin. If irisin does attenuate the glycemia that accompanies GH secretagogue treatment, then physical activity to raise the irisin level might be encouraged in patients taking these compounds. A larger study is needed to confirm this.

GH deficiency is associated with higher circulating levels of inflammation markers [10], and GH treatment inhibits the expression of proinflammatory cytokines [11, 12, 19]. In 1 study, patients with GH deficiency had increased plasma TNF-α and IL-6 levels, and treatment with GH led to a reduction in these levels [10]. In another, GH treatment in postmenopausal women with abdominal obesity reduced their serum markers of C-reactive protein and IL-6 [19]. Additionally, restoration of endogenous GH secretion after surgical removal of nonsecreting pituitary tumors was associated with a significant decline in hsCRP levels [20]. In contrast, in our study, treatment with anamorelin that restored serum IGF-1 to levels seen in young adults had no discernable impact on either IL-6 or hsCRP levels in a sample of older adults with osteosarcopenia. Similarly, we saw no indication that the IGF-1 response to anamorelin treatment was modified by the participants’ baseline circulating level of IL-6 or hsCRP. One plausible explanation for our null findings is that the inflammation level in our participants was not high enough to impair their GH/IGF-1 axis. This is plausible since we excluded participants with inflammatory diseases and insulin-dependent diabetes, and, consequently, our study participants had no more than mild to moderate levels of inflammation. Another potential explanation is that the study was too small to detect a modest effect of anamorelin treatment on inflammation markers.

Strengths of this study are that it had a randomized, placebo-controlled design, and the GH secretagogue intervention over a 1-year period significantly altered the GH/IGF-1 axis, causing a 50% increase in the circulating IGF-1 level. Limitations are that the sample size was small and the findings are not generalizable beyond older adults with osteosarcopenia. Additionally, we did not have irisin measurements at 2 months. Finally, there is wide variability in irisin assays, making it difficult to compare results across studies using different assays [21].

In conclusion, in older adults with osteosarcopenia, treatment for 1 year with the GH secretagogue anamorelin had no significant effect on irisin levels or inflammation marker levels. Higher ambient irisin levels may attenuate the glycemic response to treatment with GH secretagogues, but this requires confirmation in a larger study.

## Data Availability

The datasets generated during and/or analyzed during this study are not publicly available but are available from the corresponding author on reasonable request.

## Clinical Trial Registration

Clinicaltrials.gov: NCT 04021706
